# Inhibition of HSP70 reduces porcine reproductive and respiratory syndrome virus replication *in vitro*

**DOI:** 10.1186/1471-2180-14-64

**Published:** 2014-03-13

**Authors:** Jintao Gao, Shuqi Xiao, Xiaohong Liu, Liangliang Wang, Qianqian Ji, Delin Mo, Yaosheng Chen

**Affiliations:** 1 State Key Laboratory of Biocontrol, School of Life Sciences, Sun Yat-sen University, Guangzhou, 510006, P. R. China

**Keywords:** PRRSV, HSP70, DsRNA, Replication, Antiviral

## Abstract

**Background:**

Successful viral infection requires the involvement of host cellular factors in their life cycle. Heat shock protein 70 (HSP70) can be recruited by numerous viruses to promote the folding, maturation, or assembly of viral proteins. We have previously shown that HSP70 is significantly elevated in porcine reproductive and respiratory syndrome virus (PRRSV)-infected lungs, suggesting HSP70 may play a potential role during PRRSV infection. In this study, we tried to investigate the role of HSP70 during PRRSV infection.

**Results:**

In this study, we observed that PRRSV infection induced the expression of HSP70. The down-regulation of HSP70 using quercetin, a HSPs synthesis inhibitor, or small interfering RNAs (siRNA) reduced the viral protein level and viral production. Notably, these inhibitory effects on PRRSV infection could be attenuated by heat shock treatment. In addition, HSP70 was found to colocalize with the viral double-stranded RNA (dsRNA) and knockdown of HSP70 decreased the dsRNA levels, suggesting HSP70 is involved in the formation of viral replication and transcription complex (RTC) and thus affects the viral replication.

**Conclusions:**

Our study revealed that HSP70 is an essential host factor required for the replication of PRRSV. The inhibition of HSP70 significantly reduced PRRSV replication, which may be applied as an effective antiviral strategy.

## Background

Porcine reproductive and respiratory syndrome (PRRS) is considered to be one of the most significant viral diseases, causing serious economic losses to the swine industry worldwide [1]. The etiological agent, PRRS virus (PRRSV), is an enveloped, single-stranded positive-sense RNA virus, which is a member of the family Arteriviridae including equine arteritis virus (EAV), lactate dehydrogenase-elevating virus (LDV), and simian hemorrhagic fever virus (SHFV) [2,3]. The viral genome is approximately 15 kb in length and contains at least nine open reading frames (ORFs) [4]. The nonstructural proteins (NSPs) are encoded in ORF1a and ORF1b, which are situated in the 5’-proximal two-thirds of the genome. Some of these NSPs and host cellular factors are assembled into the double membrane vesicles (DMVs) derived from endoplasmic reticulum (ER) to form the viral replication and transcription complex (RTC) for viral replication, subgenomic (sg) mRNA transcription, and translation [4,5]. The membrane-enclosed structure in which the viral RNA synthesis takes place likely provides a stable and confined environment for replication and also protects viral RNA genome from being recognized by host response proteins [6].

Exposure of cells and tissues to extreme conditions such as heat, oxidative and osmotic stress, heavy metals, UV irradiation, microbial and viral infection leads to selective transcription and translation of heat shock proteins (HSPs) [7-10]. HSPs are highly conserved and ubiquitous cytoprotective proteins, many of which are chaperone molecules that facilitate protein folding, trafficking and also prevent their aggregation and degradation [11-13]. Based on their molecular weight, HSPs are divided into different classes: HSP100, HSP90, HSP70, HSP60, HSP40 and small HSPs [14]. As a central component of the cellular chaperone network, HSP70 is frequently recruited by numerous viruses [15]. HSP70 can be involved in different stages of the viral life cycle, including entry [16], uncoating [17], replication of the viral genome [18,19], gene expression [20] and virion morphogenesis [21,22]. Evidence is growing that HSP70 is associated with the formation of viral RTC and regulates the replication of many viruses, such as hepatitis C virus [23], flock house virus [24], herpes simplex virus type 1 [25], tomato bushy stunt tombusvirus [26]. However, the function of HSP70 during PRRSV infection has not been investigated.

We have previously shown that transcript abundance of HSP70 is elevated in PRRSV infected lungs relative to uninfected negative control (UNC) lungs [27], suggesting HSP70 may play a potential role in PRRSV infection. In this study, we aimed to investigate the role of HSP70 during PRRSV infection. Our results showed that HSP70 is up-regulated in PRRSV-infected cells. The quercetin-mediated inhibition of HSP70 expression and siRNA-mediated knockdown resulted in inhibition of viral infection. We also observed that HSP70 colocalized with the viral dsRNA generated during viral replication and knockdown of HSP70 decreased the dsRNA levels, suggesting HSP70 is involved in the formation of viral RTC and thus affects the viral replication.

## Results

### PRRSV infection induces the expression of HSP70

**Figure 1:**
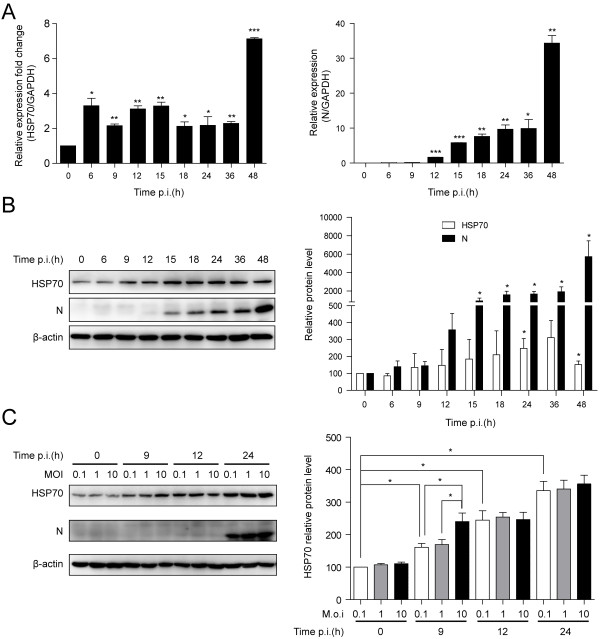
**PRRSV infection induced the HSP70 expression. (A)** MARC-145 cells were infected with PRRSV at an MOI of 0.1 and harvested at different times as indicated. The total RNA was extracted to examine the HSP70 and viral N gene mRNA levels by quantitative RT-PCR. The levels of HSP70 and N mRNA were normalized with the level of GAPDH mRNA. **(B)** Cell extracts were analyzed by Western blotting using anti-HSP70, anti-N, and anti-β-actin antibodies. β-actin served as an internal reference. The levels of HSP70 and viral N protein were quantified by measuring band intensities and normalized with respect to the amount of β-actin. **(C)** MARC-145 cells were infected with PRRSV at different MOI as indicated. Cells were harvested at different time points for Western blotting analysis. Data are mean ± SD, n = 3. *p < 0.05, **p < 0.01, ***p < 0.001.

We firstly investigated the effect of PRRSV infection on the expression of HSP70. Quantitative RT-PCR and Western blotting were performed, respectively. The level of HSP70 mRNA was up-regulated from 6 hours post infection (h.p.i) and viral N gene mRNA could be detected from 12 h.p.i (Figure 1A). The Western blotting analysis revealed that the HSP70 was induced from 9 h.p.i and viral N protein could be detected from 15 h.p.i (Figure 1B). Meanwhile, the levels of HSP70 were detected at different time points after infection with PRRSV at 0.1, 1 and 10 multiplicity of infection (MOI). The induction of HSP70 was observed (Figure 1C), which is consistent with the results shown in Figure 1B.

### Quercetin reduces the viral production

**Figure 2:**
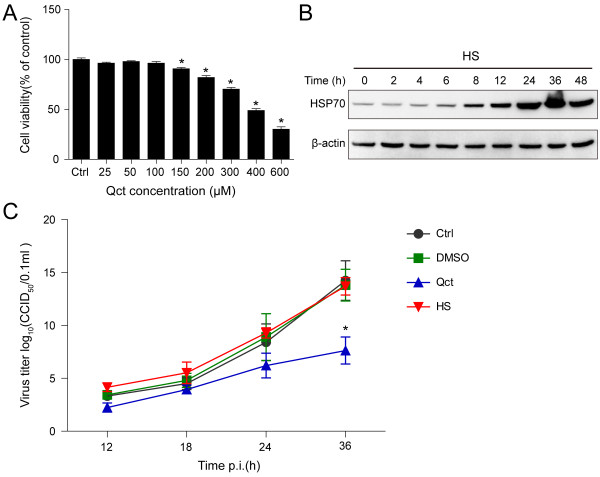
**Quercetin reduced PRRSV production. (A)** MARC-145 cells were treated with serial concentrations of quercetin (Qct) as indicated for 24 hours. Cell viability was measured with alamarBlue. Untreatment with quercetin served as control. Data are mean ± SD, n = 3, *p < 0.05 versus control (Ctrl). **(B)** MARC-145 cells were incubated at 45°C for 20 minutes and then recultured at 37°C. Cells were harvested at different times as indicated for Western blotting analysis using anti-HSP70 and anti-β-actin antibodies. **(C)** MARC-145 cells were heated (HS) or not at 45°C for 20 minutes, and 8 hours later cells were inoculated with PRRSV at an MOI of 0.1 for 1 hour. Cells without heat shock treatment were subsequently cultured with medium containing DMSO, Qct (100 μM), or no chemical (Ctrl). The culture supernatants were collected at different times and viral titers were determined by calculating CCID_50_. Data are mean ± SD, n = 3.

To investigate the function of HSP70 during PRRSV infection, we modulated the HSP70 expression by heat shock treatment or quercetin, and analyzed the effect on viral production. Quercetin had no significant toxicity on MARC-145 cells at lower concentrations (25–100 μM), but cytotoxicity was found increasing at higher concentrations (150–600 μM) (Figure 2A). Besides, the expression of HSP70 was significantly induced from 8 hours after heat shock treatment (Figure 2B). Thus, we performed the following experiments at 8 hours after heat shock treatment or with the quercetin at the concentration of 100 μM to induce or inhibit HSP70 expression, respectively. Quercetin treatment decreased the production of viral progeny at different times post infection as indicated, and remarkably at 36 h.p.i (Figure 2C, Qct). In contrast, heat shock treatment increased the viral production slightly at 12 and 18 h.p.i. However, this promotion was not found at 24 and 36 h.p.i (Figure 2C, HS).

### Quercetin prevents the viral protein expression

**Figure 3:**
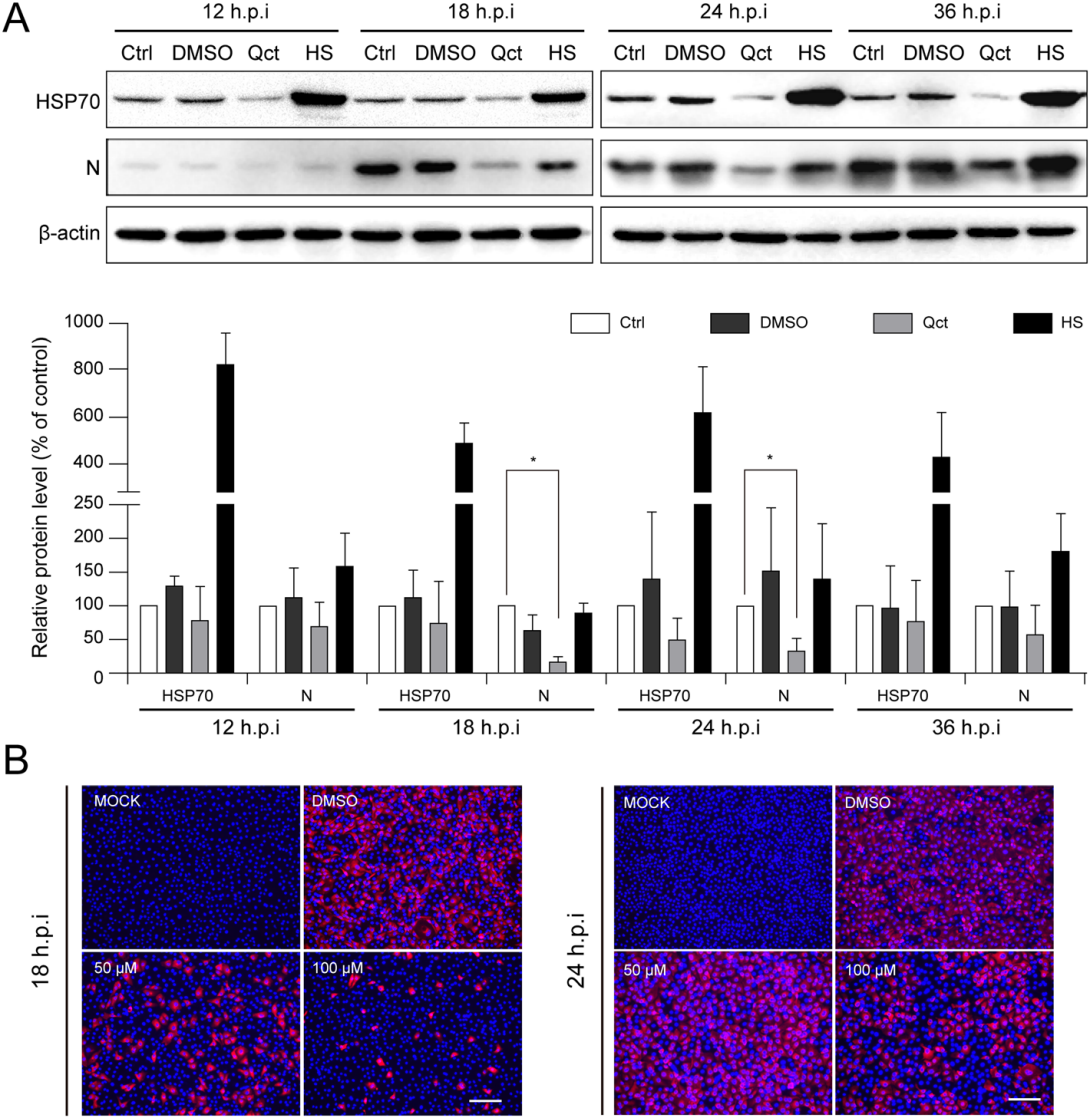
**Quercetin blocked the viral protein expression. (A)** MARC-145 cells were heated (HS) or not, and 8 hours later cells were inoculated with PRRSV at an MOI of 0.1 for 1 hour. Cells without heat shock treatment were subsequently cultured with medium containing DMSO, Qct (100 μM), or no chemical (Ctrl). Cells were harvested at different times as indicated for Western blotting analysis. The levels of HSP70 and viral N protein were quantified by measuring band intensities and normalized with respect to the amount of β-actin. Data are mean ± SD, n = 3. **(B)** PRRSV-infected MARC-145 cells were treated with DMSO or Qct at the concentration as indicated. Mock-infected cells were untreated. IFA was performed at 24 h.p.i with anti-N antibody and Alexa Fluor 555-conjugated (red) anti-mouse secondary antibody. Nuclei were stained with Hoechst dye 33258 (blue). Bar, 200 μm.

We then examined the effect of HSP70 expression on the viral protein level. Western blotting and indirect immunofluorescence assay (IFA) were performed to detect the level of viral N protein. Western blotting analysis showed that the synthesis of HSP70 was inhibited with quercetin treatment. Meanwhile, the N protein expression was found decreased at different times post infection (Figure 3A). Quercetin reduced the N protein level in a dose-dependent manner (Figure 3B). In contrast, heat shock treatment resulted in a slight increase of viral N in protein level compared to control (Figure 3A).

### Previous heat shock treatment attenuates the inhibitory effect of quercetin

**Figure 4:**
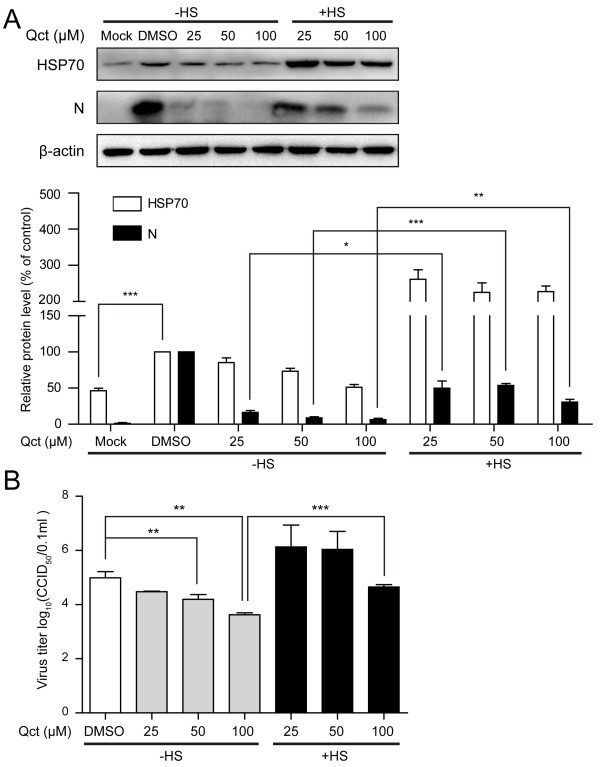
**Previous heat shock treatment attenuated the inhibitory effect of quercetin on viral infection.** MARC-145 cells were heated (+HS) or not (-HS) at 8 hours before infection. Cells were infected at an MOI of 0.1 and treated with DMSO or Qct as indicated. Mock-infected cells were untreated. **(A)** Cells were harvested at 24 h.p.i for Western blotting analysis. The levels of HSP70 and viral N protein were quantified by measuring band intensities and normalized with respect to the amount of β-actin. **(B)** The culture supernatants were collected and viral titers were determined by calculating CCID_50_. Data are mean ± SD, n = 3. *p < 0.05, **p < 0.01, ***p < 0.001.

In order to determine whether the up-regulation of HSP70 could attenuate the inhibitory effect of quercetin on PRRSV infection, MARC-145 cells were submitted to heat shock treatment and subsequently infected with PRRSV at the presence of quercetin. The results showed that quercetin reduced the N protein expression in a dose-dependent manner (Figure 4A), which is in accordance with the results showed in Figure 3B. The previous heat shock treatment could attenuate the inhibitory effect of quercetin on viral protein synthesis (Figure 4A, +HS). The effect on viral production was also analyzed. As expected, previous heat shock treatment also could attenuate the inhibitory effect on viral production (Figure 4B).

### The effect of knockdown of HSP70 on the viral infection

**Figure 5:**
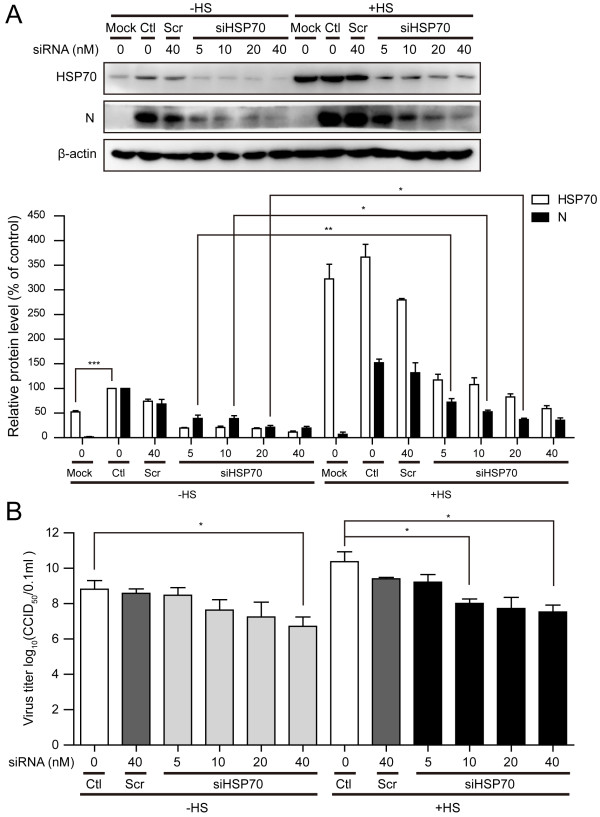
**The inhibitory effect of specific siRNA on viral infection could be rescued by heat shock treatment.** MARC-145 cells were transfected with no siRNA (Ctrl), scramble siRNA (Scr), or different concentrations of siRNAs targetting HSP70 (siHSP70). After 24 hours, cells were submitted to heat shock treatment (+HS) or not (-HS). 8 hours later, cells were mock infected or infected with PRRSV at an MOI of 0.1 and harvested at 24 h.p.i. **(A)** Cell extracts were analyzed by Western blotting. The levels of HSP70 and viral N protein were quantified by measuring band intensities and normalized with respect to the amount of β-actin. **(B)** The culture supernatants were collected and viral titers were determined by calculating by CCID_50_. Data are mean ± SD, n = 3. *p < 0.05, **p < 0.01.

Quercetin is shown to suppress the cellular levels of heat shock factor (HSF) and result in the reduction of cellular HSPs synthesis. Hence, quercetin is not a specific inhibitor of HSP70. In order to investigate the role of HSP70 during PRRSV infection more specifically, siRNA-mediated knockdown was performed. We observed that specific siRNA could inhibit the expression of HSP70 in a dose-dependent manner. Meanwhile, our results showed that the knockdown of HSP70 resulted in significant reduction of the amounts of viral N protein (Figure 5A). However, these inhibitory effects on viral N protein expression could be rescued with heat shock treatment following siRNA transfection (Figure 5A, +HS). Similar results were obtained by measuring the CCID_50_ (Figure 5B).

### The knockdown of HSP70 decreases the level of viral dsRNA

**Figure 6:**
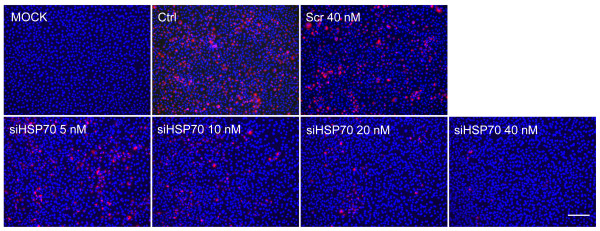
**The knockdown of HSP70 reduced the level of viral dsRNA RI.** MARC-145 cells were transfected with no siRNA (Ctrl), scramble siRNA (Scr), or different concentrations of siRNAs targetting HSP70 (siHSP70). After 24 hours, cells were mock infected or infected with PRRSV at an MOI of 0.1. Cells were fixed at 24 h.p.i and IFA was performed to detect viral dsRNA (red) with anti-dsRNA (J2) antibody. Nuclei were stained with Hoechst dye 33258 (blue). Bar, 200 μm.

The viral genome is transcribed into complementary negative-stranded RNA which in turn is used as a template to synthesize new strands forming dsRNA replicative intermedias (RIs) during positive-sense RNA virus infection. In order to investigate whether the inhibition in viral protein synthesis and viral production is due to the replication inhibition, we detected the level of the viral dsRNA generated during viral replication with specific antibody (J2). Our results showed that the level of dsRNA was reduced by siRNA in a dose dependent manner (Figure 6), suggesting HSP70 is important for PRRSV replication.

### HSP70 colocalizes with viral dsRNA

**Figure 7:**
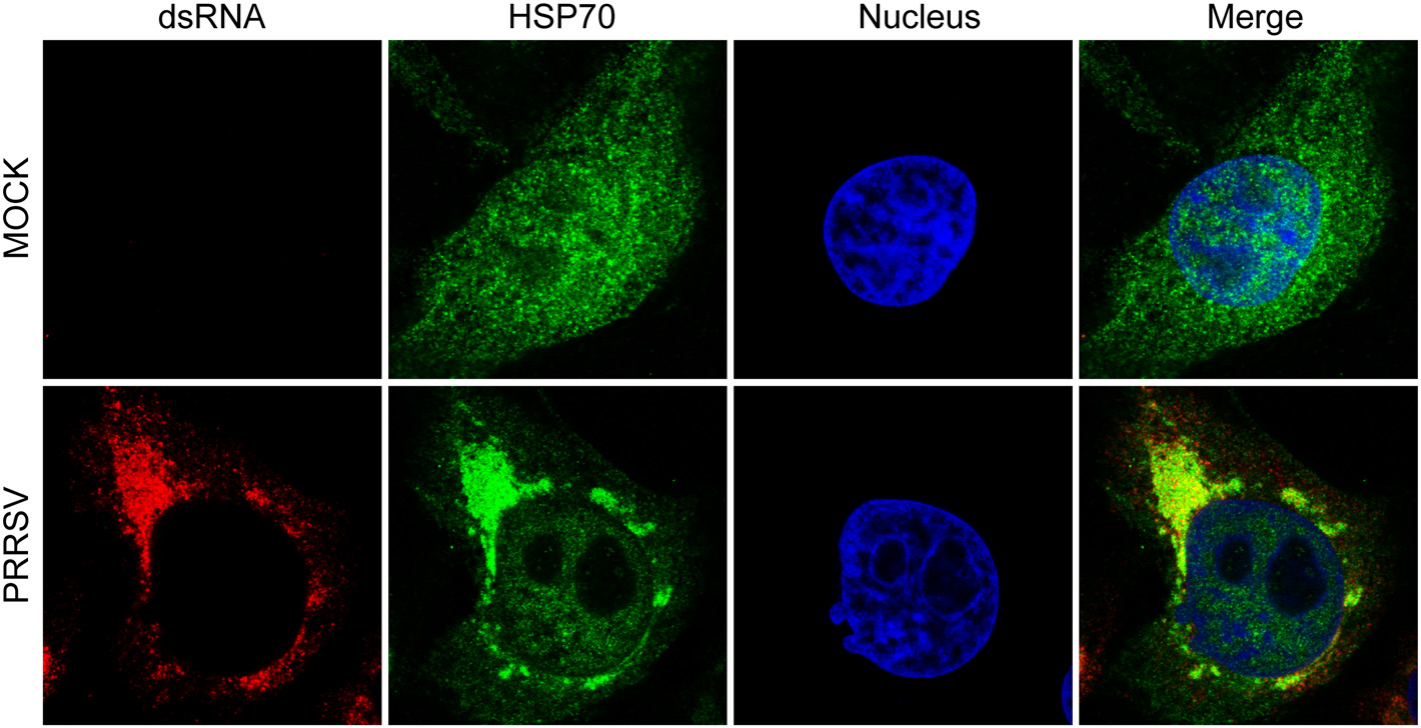
**HSP70 colocalized with viral dsRNA in infected MACR-145 cells.** MARC-145 cells were mock infected or infected with PRRSV at an MOI of 0.1. Cells were fixed at 24 h.p.i and subjected to IFA to detect dsRNA (red) and HSP70 (green) by using mouse anti-dsRNA (J2) MAb and rabbit anti-HSP70 polyclonal antibody, respectively. Nuclei were stained with Hoechst dye 33258 (blue). The images of cells were acquired with Leica TCS SP5 confocal microscope.

Positive-sense RNA virus infection generates the RTCs which contain viral and host proteins, endoplasmic reticulum (ER) membranes, as well as dsRNAs. Hence, dsRNA has been used as a marker to conveniently examine the formation of viral replication and transcription complexe (RTC) in cells infected with many positive-sense RNA viruses, such as Kunjin virus [28], rubella virus [29], hepatitis C virus [6] and PRRSV [5]. The knockdown of HSP70 reduced the amount of PRRSV dsRNA (Figure 6), implying that HSP70 may be involved in the formation of RTC. To understand the relationship between HSP70 and viral RTC, confocal microscopy was performed to detect HSP70 and dsRNA. Our results showed that HSP70 was detected as diffuse staining in both the nuclear and cytoplasmic regions in uninfected cells, while it was found mostly accumulating in the perinuclear region and colocalizing with dsRNA in PRRSV-infected MARC-145 cells (Figure 7).

### Quercetin reduced PRRSV infection in porcine alveolar macrophages

**Figure 8:**
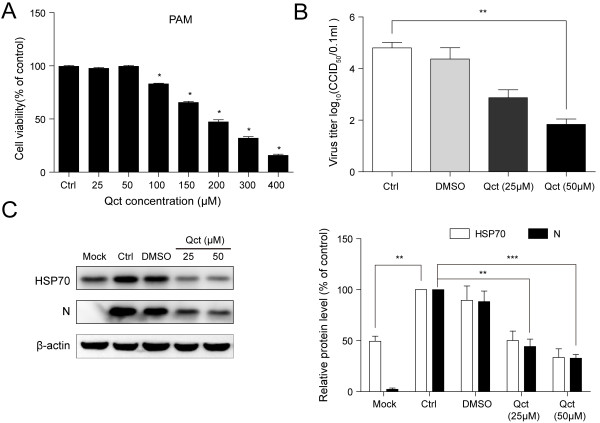
**Quercetin reduced PRRSV infection in PAMs. (A)** PAMs were treated with serial concentrations of Qct as indicated for 24 hours. Cell viability was measured with alamarBlue. Untreatment with quercetin served as control. Data are mean ± SD, n = 3, *p < 0.05 versus control (Ctrl). **(B)** PRRSV-infected PAMs were infected with PRRSV at an MOI of 0.1 for 1 hour and subsequently treated with Qct at the concentration as indicated. Mock-infected cells were untreated. The culture supernatants were collected at 24 h.p.i and viral titers were determined by calculating by CCID_50_. **(C)** Cells were harvested for Western blotting analysis. The relative protein level was quantified by measuring band intensities and normalized with respect to the amount of β-actin. Data are mean ± SD, n = 3. **p < 0.01, ***p < 0.001.

Porcine alveolar macrophages (PAMs) are known to be an important primary target for PRRSV replication, thus the anti-PRRSV effect of quercetin was evaluated in PAMs. PAMs were more sensitive to quercetin compared to MARC-145 cells, and the minimal toxicity was found at concentration below 50 μM (Figure 8A). Similar inhibitory effect on PRRSV infection was found in PAMs with quercetin treatment (Figure 8B and C). Notably, HSP70 was induced in PRRSV-infected PAMs (Figure 8C), which is consistent with the observation shown in Figure 1.

## Discussion

PRRSV infection results in substantial economic losses to the swine industry worldwide. However, no effective countermeasures exist to combat this deadly viral infection so far. The identification of host factors and exploration of their functions during virus infection not only will enable greater insight into the molecular mechanisms of viral pathogenesis, but also will provide a potential for the development of antiviral strategies.

Virus infection leads to changes of many host proteins expression, and up-regulation of HSP70 following viral infection has been widely observed [15]. Recently, HSP70 was also found to be elevated after PRRSV infection based on transcriptome and proteome approaches [27,30,31]. In this study, we observed that PRRSV infection induced HSP70 expression *in vitro* (Figures 1 and 8C), implying that HSP70 may play a potential role in PRRSV infection.

Virus-induced HSP70 could be utilized to facilitate viral infection or to enhance intracellular defense against the invading microorganism. Hence, HSP70 can regulate the viral infection positively or negatively [32-34]. To better understand the role of HSP70 during PRRSV infection, we modulated the expression of HSP70 and analyzed the effect on viral infection. We observed that the down-regulation of HSP70 significantly reduced the level of viral N protein and viral production (Figures 2C, 3, 4, 5 and 8). PAMs are known to be the primary host cellular target for PRRSV replication, thus the significant anti-PRRSV effect of quercetin in these cells (shown in Figure 8) suggests that it might also be effective agent against PRRSV infection *in vivo*. However, overexpression of HSP70 following heat shock treatment resulted in slight increase of viral protein level and viral production (Figures 2C, 3A and 5), which is consistent with a previous research [32]. This is likely due to the fact that PRRSV infection induced a rather high level of HSP70, which is sufficient to support PRRSV replication.

As expected, previous heat shock treatment could attenuate the inhibitory effects of quercetin on the PRRSV (Figure 4). Notably, quercetin at the concentration of 100 μM still has a strong inhibitory effect even with the previous heat shock treatment (Figure 4). This is likely because that quercetin at higher concentration powerfully inhibits the HSPs protein synthesis, and up-regulation of inducible HSPs (including HSP70) induced by previous heat shock treatment can not compeletly compensate the inhibition effect of quercetin. These results suggested other chaperones which are generally constitutive and not sensitive to stimuli may also be involved in the PRRSV life cycle, such as heat shock cognate protein 70 (HSC70) and HSP90β [35,36]. HSC70 can be involved in different steps of viral life cycle, such as entry [37,38], disassembly [39], translocation [40] and release [41]. HSP90β, a constitutive cytoplasmic isoform of HSP90, has been reported as a critical host factor required for Japanese encephalitis virus (JEV) infectivity in BHK-21 cells [42]. Further studies may be required to address whether HSC70 and HSP90β are involved in the PRRSV life cycle and to figure out their role during viral infection.

Using siRNA-mediated silencing approach, we specifically established the importance of HSP70 during PRRSV infection. We observed that siRNA-mediated depletion of HSP70 led to inhibition of viral protein synthesis and viral production in a dose-dependent manner. However, this inhibition could be rescued by heat shock treatment following transfection (Figure 5). These results indicated that HSP70 is essential for PRRSV infection, suggesting its proviral nature.

Positive-sense RNA virus infection forms dsRNA RI following the synthesis of complementary negative-sense RNA which is used as template to synthesize new strands. To investigate whether HSP70 play any role in the PRRSV replication, we detected the dsRNA level using specific antibody (J2). Our results showed that the knockdown of HSP70 resulted in reduction of dsRNA (Figure 6), indicating HSP70 is important for PRRSV replication. Viral dsRNA is contained in the RTC, hence dsRNA is used as a marker to examine the formation of viral RTC [5,6,28,43,44]. The reduction of dsRNA level may be due to the blockade of viral RTC formation. Confocal microscopic analysis was performed to examine if HSP70 associates with the RTC. We observed a strong colocalization of cytoplasmic HSP70 and dsRNA in PRRSV-infected cells (Figure 7), suggesting HSP70 may be involved in the formation of viral RTC and thus affect the viral replication. The formation of RTC composed of viral dsRNA RIs, viral replicases, altered cellular membranes and some cellular proteins, is a hallmark of all positive-stranded RNA viruses [23]. HSP70 is frequently recruited to help the assembly of viral replicases into the RTC [23-26,45]. Previous studies have implicated that several replicases of PRRSV, including NSP1β, NSP2, NSP3, NSP4, NSP7α, NSP7β, NSP8 and NSP9 may be included in the PRRSV RTC [4,5,46]. HSP70 may be recruited to enhance these NSPs stability and to assist their translocation into the RTC. Further studies are required to address the interactions of HSP70 with these NSPs, and to figure out how these interactions might regulate viral replication.

## Conclusions

In conclusion, our study has demonstrated that HSP70 is a crucial host factor recruited by PRRSV and plays a positive role in regulating the viral replication. Furthermore, our findings suggested that inhibition of HSP70 might be an efficient antiviral strategy against PRRSV infection. The direct interactions of HSP70 and viral proteins should be determined in details in the future.

## Methods

### Cell culture and virus infection

MARC-145 cells were cultured in Dulbecco’s modified Eagle’s medium (DMEM) containing 10% Fetal Bovine Serum (FBS) and maintained at 37°C with 5% CO_2_. PAMs were obtained postmortem lung lavage of 8-week-old specific pathogen free pigs, and maintained in RPMI 1640 medium containing 10% FBS and penicillin/streptomycin. Cells were infected with PRRSV strain CH-1a (the first type 2 PRRSV strain isolated in China, kindly provided by Dr. Guihong Zhang in South China Agricultural University, China). Virus titers were determined by calculating 50% cell culture infectious dose (CCID_50_) using the Reed-Muench method.

### Antibodies

The mouse anti-HSP70 MAb (SPA-810) and rabbit polyclonal anti-HSP70 antibody (SPA-812) were obtained from Enzo Life Sciences (Farmingdale, NY, USA), and the rabbit anti-β-actin MAb (13E5) was obtained from Cell Signaling Technology (Beverly, MA, USA). The anti-PRRSV N protein MAb was obtained from Jeno Biotech Inc (Chuncheon, South Korea). The mouse monoclonal antibody (J2) specific for dsRNA was purchased from Scicons (Hungary).

### Heat shock and quercetin treatment

To induce the expression of HSP70, MARC-145 cells were heated at 45°C for 20 minutes and then recultured at 37°C. After 8 hours, cells were inoculated with PRRSV for 1 hour. The medium containing DMSO or different amounts of quercetin (Sigma) was added.

### Cell viability assay

Cells were seeded into 96-well plates and treated with quercetin for 24 hours. The medium then was exchanged with fresh medium containing 10% alamarBlue (Invitrogen, Carlsbad, CA, USA) for 4 hours according to the manufacturer’s instruction. The fluorescence was monitored at 570 nm excitation and 590 nm emission wavelengths and was directly proportional to the number of living cells.

### SiRNAs and transfection

SiRNAs obtained from Ribobio (Guangzhou, China) were designed to interfere with the two mRNAs encoding HSP70 (GenBank accession number AB170713 for HSPA1A and XM_001115060 for HSPA1B). MARC-145 cells were seeded into 6-well plates nearly 24 hours before tansfection. The siRNAs were transfected into MARC-145 cells with lipofectamine 2000 (Invitrogen) according to the manufacturer’s instruction.

### Quantitative RT-PCR assays

Total RNA was isolated from PRRSV-infected MARC-145 cells using TRIzol™ reagent (Invitrogen). Reverse transcription was carried out using Reverse Transcription System (Promega, Madison, WI, USA) according to the manufacturer’s instruction. Quantitative PCR was performed in LightCycler® 480 Real-Time PCR System (Roche). Amplification was carried out in a 10 μl reaction mixture containing 5 μl SYBR® Premix Ex Taq™ (TaKaRa, China), 0.2 μM concentration of each primer, and 1 μl cDNA. The reaction procedure was 95°C for 10 seconds, followed by 40 cycles at 95°C for 5 seconds and 60°C for 40 seconds. GAPDH served as an internal reference. Specific primers were used for the amplification of HSP70, GAPDH or viral N genes: HSP70-F,5′-AGGAGTTCCATATCCAGAA-3′; HSP70-R,5′-CAGCTCGACATTCACCAC-3′; GAPDH-F,5′-TGACAACAGCCTCAAGATCG-3′; GAPDH-R,5′-GTCTTCTGGGT-GGCAGTGAT-3′;N-F,5′-AAAACCAGTCCAGAGGCAAG-3′;N-R,5′-CGGATCA-GACGCACAGTATG-3′.

### Western blotting

Cells were washed with PBS, lysed in cell lysis buffer (Beyotime Biotechnol, Shanghai, China) containing 1 mM phenylmethyl-sulfonylfluoride (PMSF) and boiled for 5 minutes. About 25 μg of protein was subjected to 12% sodium dodecyl sulfate-polyacrylamide gel electrophoresis (SDS-PAGE), followed by blotting onto a polyvinyl difluoride (PVDF) membrane. After blotting, the membrane was blocked in 0.05% TBS-Tween (TBST) containing 5% nonfat dry milk for 2 hours and incubated overnight at 4°C with primary antibody. After being washed three times in TBST, the membrane was incubated with the HRP-conjugated secondary antibody for 1 hour at room temperature. Visualisation was performed with chemiluminescence substrate (Pierce, IL, USA) using Image Station 4000 mm PRO System (Kodak). Protein band intensities were measured by using Image Station 4000 mm PRO software.

### Indirect immunofluorescence assay

Cells grown on coverslips were washed with PBS, and then fixed with 4% paraformaldehyde for 10 minutes at room temperature. After three times washes in PBS, the cells were then permeabilized with 0.5% Triton X-100 for 15 minutes and blocked in PBS containing 1% bovine serum albumin (BSA) for 30 minutes at room temperature. The coverslips were then incubated overnight with primary antibodies in PBS containing 1% BSA at 4°C. After being washed three times with PBS, the coverslips were incubated with Alexa Fluor 555-labeled anti-mouse or Alexa Fluor 488-labeled anti-rabbit secondary antibodies (CST) for 1 hour at room temperature. After three times washes, the Hoechst dye 33258 (Sigma) was added to stain the nuclei. After staining for 4 minutes, the coverslips were washed, followed by mounting onto the microscope slides, and observed using a Zeiss ELYRA P.1 microscope or a Leica TCS SP5 confocal microscope.

### Statistical analysis

Results were means ± standard errors of three independent experiments. Statistical significance was determined by Student’s *t* test. Differences were considered to be statistically significant for p values < 0.05.

## Competing interests

The authors declare that they have no competing interests.

## Authors’ contributions

JG and SX conceived and designed the study. JG performed the experiments, analyzed the data, and wrote the manuscript. SX, XL, LW, QJ, DM coordinated the study. YC contributed to the interpretation of the results and took part in the critical revision of the manuscript. All authors read and approved the final manuscript.
